# Single-cell sequencing in stem cell biology

**DOI:** 10.1186/s13059-016-0941-0

**Published:** 2016-04-15

**Authors:** Lu Wen, Fuchou Tang

**Affiliations:** Biodynamic Optical Imaging Center, College of Life Sciences, Peking University, Beijing, 100871 China; Peking-Tsinghua Center for Life Sciences, Peking University, Beijing, 100871 China

## Abstract

Cell-to-cell variation and heterogeneity are fundamental and intrinsic characteristics of stem cell populations, but these differences are masked when bulk cells are used for omic analysis. Single-cell sequencing technologies serve as powerful tools to dissect cellular heterogeneity comprehensively and to identify distinct phenotypic cell types, even within a ‘homogeneous’ stem cell population. These technologies, including single-cell genome, epigenome, and transcriptome sequencing technologies, have been developing rapidly in recent years. The application of these methods to different types of stem cells, including pluripotent stem cells and tissue-specific stem cells, has led to exciting new findings in the stem cell field. In this review, we discuss the recent progress as well as future perspectives in the methodologies and applications of single-cell omic sequencing technologies.

## Background

An individual cell is the smallest functional and universal unit of organisms. Gene expression is regulated within or between individual cells, and so, ideally, analyses of gene expression would be performed using single cells; but owing to technical limitations, such as the tiny size of an individual cell, nearly all of the gene-expression studies described in the literature (especially those at a whole-genome scale) have been performed using bulk samples of thousands or even millions of cells. The data based on these ensemble analyses are valid; but the gene expression heterogeneity between individual cells, especially at the whole-genome scale, is still largely unexplored.

Cellular heterogeneity is a general feature of biological tissues that is influenced by both physiological and pathological conditions. Even a ‘pure’ cell type will have heterogeneous gene expression because individual cells may occur in a range of extrinsic microenvironments and niches that influence gene expression, because gene expression may differ throughout the cell cycle, and because of the intrinsic stochastic nature of gene-expression systems [1–4]. By definition, a stem cell is characterized as both being capable of unlimited self-renewal and having the potential to differentiate into specialized types of cells. Stem cells are generally classified into pluripotent stem cells, which can give rise to cells of all three germ layers (the ectoderm, mesoderm and endoderm), and tissue-specific stem cells, which play essential roles in the development of embryonic tissues and the homeostasis of adult tissues. Pluripotent stem cells in a mammalian early embryo are few in number; tissue-specific stem cells always form a minor proportion of the cell population of a particular tissue or organ. These minor cell populations are thus intermingled with a variety of differentiated and intermediate cell types in the embryonic or adult tissues, forming heterogeneous populations. Single-cell sequencing provides powerful tools for characterizing the omic-scale features of heterogeneous cell populations, including those of stem cells. The beauty of single-cell sequencing technologies is that they permit the dissection of cellular heterogeneity in a comprehensive and unbiased manner, with no need of any prior knowledge of the cell population.

In this review, we discuss the methodologies of recently developed single-cell omic sequencing methods, which include single-cell transcriptome, epigenome, and genome sequencing technologies, and focus on their applications in stem cells, both pluripotent and tissue-specific stem cells. Finally, we briefly discuss the future of methodologies and applications for single-cell sequencing technologies in the stem cell field.

## Single-cell RNA-sequencing (RNA-seq) technologies

### Introduction of single-cell RNA-seq technologies

RNA-seq technology provides an unbiased view of the transcriptome at single-base resolution. It has been shown that the transcriptome of a mammalian cell can accurately reflect its pluripotent or differentiated status, and it will be of great interest to explore the transcriptome diversity and dynamics of self-renewing and differentiating stem cells at single-cell resolution. The first method for single-cell RNA-seq was reported in 2009, only 2 years after standard RNA-seq technology using millions of cells was developed [5]. Subsequently, many other single-cell RNA-seq methods based on different cell capture, RNA capture, cDNA amplification, and library establishment strategies were reported, including Smart-seq/Smart-seq2 [6, 7], CEL-seq [8], STRT-seq [9, 10], Quartz-seq [11], multiple annealing and looping-based amplification cycles (MALBAC)-RNA [12], Phi29-mRNA amplification (PMA), Semirandom primed polymerase chain reaction (PCR)-based mRNA amplification (SMA) [13], transcriptome in vivo analysis (TIVA) [14], fixed and recovered intact single-cell RNA (FRISCR) [15], Patch-seq [16, 17], microfluidic single-cell RNA-seq [18, 19], massively parallel single-cell RNA-sequencing (MARS-seq) [20], CytoSeq [21], Drop-seq [22] and inDrop [23].

Methods allowing in situ single-cell RNA sequencing or highly multiplexed profiling have also been developed recently [24, 25]. Furthermore, methods for three-dimensional reconstructed RNA-seq at single-cell resolution have also been developed [26–28]. A summary of these methods can be found in Table 1, and detailed descriptions of them can also be seen in other recent reviews [29–31]. All of these methods detect only poly(A)-plus RNAs from an individual cell and thus miss the important poly(A)-minus RNAs. Recently, we developed the SUPeR-seq technique, which detects both poly(A)-plus and poly(A)-minus RNAs from an individual cell, and we used it to discover several thousands of circular RNAs with no poly(A) tail as well as hundreds of poly(A)-minus linear RNAs in mouse pre-implantation embryos [32].

**Table 1:** Summary of single-cell RNA-seq technologies

Assays	Cell capture strategies	cDNA amplification strategies	Target RNAs	Poly(A) minus RNA detection	Number of cells	UMI	Reference(s)
scRNA-seq	Mouth pipette or FACS	Polyadenylation followed by PCR	Full-length mRNAs	No	1–100	No	[5]
Quartz-seq	Mouth pipette or FACS	Polyadenylation followed by PCR	Full-length mRNAs	No	1–100	No	[11]
Smart-seq/Smart-seq2	Mouth pipette or FACS	Template-switch followed by PCR	Full-length mRNAs	No	1–100	No	[6, 7]
MALBAC-RNA	Mouth pipette or FACS	MALBAC	Full-length mRNAs	No	1–100	No	[12]
PMA	Mouth pipette or FACS	Rolling circle amplification	Full-length mRNAs	No	1–100	No	[13]
SMA	Mouth pipette or FACS	Semi-random priming followed by PCR	Full-length mRNAs	No	1–100	No	[13]
SUPeR-seq	Mouth pipette or FACS	Random priming followed by PCR	Full-length mRNAs	Yes	1–100	No	[32]
Fluidigm C1	Microfluidic system	Template-switch followed by PCR	Full-length mRNAs	No	100–1000	No	[18]
Microfluidic scRNA-seq	Microfluidic system	Polyadenylation followed by PCR	Full-length mRNAs	No	100–1000	No	[19]
STRT-seq	Mouth pipette or FACS	Template-switch followed by PCR	5′ end of mRNAs	No	10–100	Yes	[9, 10]
CEL-seq	Mouth pipette or FACS	In vitro transcription	3′ end of mRNAs	No	10–100	Yes	[8]
MARS-seq	Robotics and automation	CEL-seq	3′ end of mRNAs	No	100–1000	Yes	[20]
CytoSeq	Bead-based	CEL-seq	3′ end of mRNAs	No	>1000	Yes	[21]
Drop-seq	Droplet- and bead-based	Template-switch followed by PCR	3′ end of mRNAs	No	>1000	Yes	[22]
inDrop	Droplet- and bead-based	CEL-seq	3′ end of mRNAs	No	>1000	Yes	[23]
TIVA	In vivo mRNA capture based on photo-activation	In vitro transcription	Full-length mRNAs	No	10–100	No	[14]
FRISCR	FACS or fixed cells	SMART-seq2	Full-length mRNAs	No	10–100	No	[15]
Patch-seq	Aspiration through patch-clamp pipette	STRT-seq/SMART-seq2	5′ end of mRNAs or full-length mRNAs	No	10–100	Yes/no	[16, 17]
FISSEQ	In situ RNA sequencing	Rolling circle amplification	Full-length mRNAs	No	100–1000	No	[24]

*FACS* fluorescence-activated cell sorting, *FISSEQ* fluorescence in situ sequencing, *FRISCR* fixed and recovered intact single-cell RNA, *MALBAC* multiple annealing and looping-based amplification cycles, *MARS* massively parallel single-cell RNA-sequencing, *PCR* polymerase chain reaction, *PMA* Phi29-mRNA amplification, *sc* single-cell, *seq* sequence, *SMA* semirandom primed PCR-based mRNA transciptome amplification, *STRT-seq* single-cell tagged reverse transcription, *TIVA* transcriptome in vivo analysis, *UMI* unique molecular identifier

To obtain a comprehensive view of the heterogeneity of a complex population of cells, a large number of individual cells must be sequenced. During the past several years, the throughput of the single-cell RNA-seq technologies has been greatly improved. The microfluidic and robotic systems provide high-throughput strategies that can handle hundreds of individual cells [18–21]. Notably, two recently reported methods, Drop-seq and inDrop, dramatically improve the throughput to thousands or even tens of thousands of individual cells for each experimental run by using a combination of the one-bead–one-cell droplet and an unique barcoding strategy [22, 23]. Very different cell types can be distinguished by sequencing as few as 50,000 reads for each cell [33, 34], though deeper sequencing may be necessary to discriminate between types of cells that have relatively subtle differences, such as mouse embryonic stem cells and epiblast stem cells.

Many bioinformatics tools that were designed for bulk RNA-seq analyses are also applicable to single-cell RNA-seq data; further tools have been designed specifically for analyses of single-cell RNA-seq data. An in-depth review of these approaches can be seen elsewhere [35]. Bioinformatics tools have been used in the stem cell field to identify different cell types and sub-populations, as well as their marker genes, from the relatively noisy dataset. Determining sub-populations of stem cells within a dataset is achieved by methods for unbiased clustering and differential gene expression analysis. Zeisel et al. [36] recently described a biclustering-based algorithm called BackSPIN that increases the accuracy of identifying cell types from single-cell RNA-seq data. Grun et al. [37] developed another algorithm called RaceID, which is based on a feature of the single-cell RNA-seq technique that creates extremely low false-positive errors if cross contamination is carefully controlled, especially when unique molecular identifiers (UMIs) are applied. It does, however, generate a high number of false negative errors, where a gene is expressed in a cell, but missed by this technique. These and other methods have greatly improved the analyses of single-cell RNA-seq data in stem cells or embryos. In addition, bioinformatic analysis algorithms such as Monocle and Waterfall have been developed to provide a time-serial reconstruction of a developmental or differentiation process, also using single cell RNA-seq datasets [38, 39]. These algorithms produce a ‘pseudotime’ trajectory through a reduced dimension data space by calculating a minimum spanning tree.

Quantitative assessment of the current single-cell RNA-seq methods shows that these methods have a capture efficiency ranging between 5 % and 60 % [10, 18, 19, 40, 41]. Owing to the biases of molecular capture and amplification, current methods for the sequencing of single cells still have relatively high technical noise, which is acceptable when studying highly expressed genes but which masks the biological variations of genes that are expressed at low levels. Several studies have made great efforts to improve signal-to-noise performance by optimizing the efficiency of reverse transcription and PCR amplification [7], by performing the reactions in nanoliter volumes in a microfluidic system instead of in microliter volumes in tubes [18, 19], through the use of UMIs [10, 33], or by using spike-in of reference mRNAs to discriminate the technical noise and real biological variation signals [42]; nevertheless, there is still much room for improvement.

**Fig. 1:**
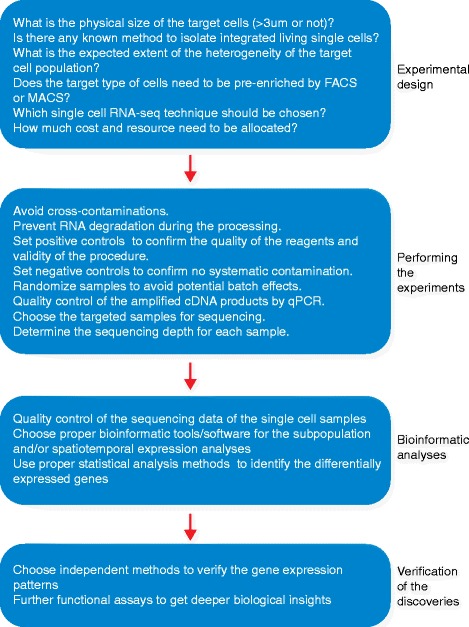
Flowchart of a typical single cell omic sequencing project. A typical single-cell sequencing project comprises four major steps: experimental design, performing the experiments, bioinformatic analyses and verification of the discoveries. Here we use a single-cell RNA-seq project as an example. Note that if the project fails at any step, researchers should go back to previous steps to identify the cause of the failure and re-design accordingly. In a real project, this process may need to be repeated several times. *FACS* fluorescence-activated cell sorting, *MACS* magnetic-activated cell sorting, *qPCR* quantitative polymerase chain reaction

In the past several years, single-cell RNA-seq methods have been applied to a wide variety of systems, including early mammalian embryos [43–48], developing tissues [33, 49–51], adult tissues [22, 36, 37, 52, 53], immune cells [20, 21, 54–56], cancer cells [6, 57–59], and stem cells that are either isolated in vivo [39, 60–63] or cultured in vitro [23, 38, 64–67]. A flowchart of a typical single-cell RNA-seq project is shown in Fig. 1. The work of Zeisel et al. is an excellent and representative example of these studies, showing that single-cell RNA-seq can identify numerous sub-populations of cells that would be missed if bulk RNA-seq were performed instead [36]. These authors unbiasedly sequenced the transcriptomes of 3005 single cells isolated from the mouse primary somatosensory cortex (S1) and the hippocampal CA1 region. A total of 47 molecularly distinct subclasses of cells were identified, comprising nine major cell types including S1 and CA1 pyramidal neurons, interneurons, oligodendrocytes, astrocytes, microglia, vascular endothelial cells, mural cells, and ependymal cells. This and other studies demonstrate that the current single-cell RNA-seq technology, even with much room for improvement, has become an established and powerful tool that has practical applications in a wide variety of biological fields.

### Pluripotent stem cells

#### Pre-implantation development

Mammalian pre-implantation development represents the start of a new life and involves global gene expression changes during this process. Because the cell numbers during this developmental process are very limited, single-cell RNA-seq provides an unprecedented opportunity to decipher gene expression dynamics during this process. Comprehensive sets of transcriptome profiles from both human and mouse cells undergoing pre-implantation development have been generated [43–45]. The gene expression features of the maternal-zygotic transition have been accurately captured. Although cells of the same stage are relatively similar, there is evidence that inter-blastomere differences occur as early as the four-cell stage of mouse embryos [46, 68]. These differences may be functionally relevant to the first cell-fate decision event of the pre-implantation embryo, which is the segregation between the trophectoderm (TE) and the inner cell mass (ICM). Later, the ICM further segregates into primitive endoderm (PE) and pluripotent epiblast (EPI) that give rise to all the cell lineages of the embryo proper. Single-cell RNA-seq analysis offers a comprehensive view of the transcriptome of these divergent cell lineages. It has been shown that *Id2* and *Sox2* are two early markers that are strongly activated in TE and ICM cells, respectively, during the 16- to 32-cell stage of the mouse embryo [69]. In addition, before the segregation of PE and EPI, a precursor cell expresses both the PE and EPI markers, leading to a model of stochastic cell-to-cell expression heterogeneity that is followed by signal reinforcement and commitment of cell-fate determination [70].

Conservation of gene expression and differences between human and mouse pre-implantation development have been identified on the basis of single-cell RNA-seq data. One documented difference between human and mouse development is the timing of zygotic genome activation, which has been clearly verified using single-cell transcriptome data. In a mixed background mouse (CAST/EiJ × C57BL/6 J), embryos showed rapid maternal transcript clearance and zygotic genome activation at the two-cell stage, as well as significant allele-specific gene expression [45]. In humans, principal component analysis (PCA) and differential gene expression analysis confirmed that zygotic genome activation occurs between the four- and eight-cell stages [44]. A careful comparative analysis revealed many other important differences between human and mouse development [47]. For example, the transcription factor *KLF17* is exclusively expressed in the human EPI, and key components of the transforming growth factor (TGF)-β signaling pathway are highly enriched in human, but not mouse, embryos. In addition, the key factors *Id2*, *Elf5*, and *Eomes* are exclusively expressed in TE cells in the mouse, but not in humans.

#### Embryonic stem cells

Both mouse and human embryonic stem cells (ESCs) serve as excellent in vitro models for studying the self-renewal ability and differentiation potential of pluripotent stem cells. The ICM of blastocysts can form ESCs when cultured in proper pluripotency maintenance conditions, and the derivation of both human and mouse ESCs have been traced using single-cell RNA-seq methods [44, 64]. These studies demonstrate that the outgrowth process is associated with prominent expression changes for transcriptional regulators and for genes that are associated with pluripotency. A comparison between human embryonic stem cells (hESCs) and EPI showed that genes that are involved in pluripotency are conserved, but enriched for different pathways [44, 47]. Human EPI is enriched for oxidative phosphorylation signaling, reflecting a difference in growth environment from that of hESCs, which are cultured in vitro under oxygen-rich conditions and preferentially switch to glycolytic metabolism. The hESCs are enriched for the regulation of cell proliferation and genes involved in the fibroblast growth factor (FGF), MAPK and Wnt signaling pathways, suggesting that the EPI and hESCs have distinct mechanisms for maintaining the pluripotency state.

Although ESCs are relatively homogeneous, they still contain different sub-populations. Single-cell RNA-seq analysis has revealed that many genes have variable expression among individual mouse embryonic stem cells (mESCs) [18, 19] and, importantly, has identified sub-populations that have distinct transcriptomes [23, 65, 66]. By sequencing nearly 1000 individual mESCs using the droplet-barcoding approach, Klein et al. [23] characterized several minor sub-populations, including an epiblast-like sub-population, a Prdm1-high sub-population and an Hsp90-high sub-population. The same study also sequenced thousands of cells to examine the differentiation of mESCs after withdrawal of leukemia inhibitory factor (LIF), and characterized the dynamic changes during differentiation in several sub-populations that do not map to any known cell type.

#### Primordial germ cells

Primordial germ cells (PGCs) are precursors of mature germ cells—the oocyte and sperm. Single-cell RNA-seq datasets of human PGCs from the migrating stage to the gonadal stage have been created and reveal the dynamic and balanced expression of both pluripotency genes and germline-specific genes during PGC development [62]. Cell populations of early PGCs in mitosis are relatively homogenous, whereas the later female PGCs are highly heterogeneous during meiotic arrest, even within the same embryo. This finding suggests that entry into meiotic arrest is unsynchronized for human female PGCs in vivo. Unique features that distinguish human PGCs from those of mice were also systematically explored. For example, human early PGCs highly expressed *SOX15* and *SOX17*, whereas those from mice express *Sox2*.

### Tissue-specific stem cells

Tissue-specific stem cells reside in developing or differentiated tissues. They also undergo self-renewal and have the potential to differentiate into a variety of specified cell types. In the past 2 years, single-cell RNA-seq methods have been applied to tissue-specific stem cells. These studies have identified novel stem cell types and have dissected cell heterogeneity within a ‘homogenous’ stem cell population.

#### Identification of novel stem cell types

The study by Treutlein et al. [49] on developing mouse lung epithelium provides an elegant example of how a novel stem cell type could be identified using the single-cell RNA-seq approach. The alveolar type 1 (AT1) and AT2 cells in the lung are two epithelial cell types that play crucial roles in air exchange, but the identity of the alveolar progenitors remains elusive. Treutlein et al. [49] identified five distinct cell populations through evaluation of 80 individual epithelial cells from distal lung regions of E18.5 mouse embryos, which included four known cell types: two bronchiolar lineages (Clara and ciliated cells), and the alveolar type AT1 and AT2 cells. An undefined and interesting fifth cell group co-expresses the marker genes of AT1 and AT2 and is positioned between the populations of AT1 and AT2 cells on the PCA plot, pointing to a bi-potential progenitor population for AT1 and AT2 cells. These alveolar progenitors have been verified by an independent set of experiments, including immunostaining, lineage tracing and clonal analysis [71]. Furthermore, single-cell RNA-seq data allowed Treutlein et al. [49] to characterize the developmental intermediates from the bi-potential progenitor to AT1 and AT2 cells completely, and even to reconstruct a continual route of the differentiation process to reveal the transcriptome dynamics.

#### Dissecting cell heterogeneity among a stem cell population

Single-cell RNA-seq has been used to dissect cellular heterogeneity within a tissue-specific stem cell population. These studies have revealed both similarities and differences in the structures of the stem cell populations of different tissue types. Hematopoietic stem cells (HSCs) generate all blood lineages. Long-term reconstituting HSCs (LT-HSCs) are at the top of the hematopoietic hierarchy and can undergo self-renewal and division to replenish committed cells, which are called short-term reconstituting HSCs (ST-HSCs). Kowalczyk et al. [60] and Tsang et al. [61] have shown that cell-cycle differences dominate the cell heterogeneity of each HSC type. The cell-cycle progression of HSCs can be re-established using single-cell transcriptome data, which provide a promising new approach for studying the characteristics of quiescent and proliferative stem cells. Analysis of non-cycling cells revealed a clear difference between LT-HSCs and ST-HSCs. Within the LT-HSCs, subgroups of cells that are associated with markers of specific lineages also exist, as revealed by analyzing only the hematopoietic genes, even though these genes may still be related to the cell cycle.

The neural stem cells (NSCs) in the subventricular zone and the subgranular zone of the dentate gyrus continually give rise to new neurons and glia in the adult mammalian brain. The neurogenesis process starts from quiescent NSCs (qNSCs), which become activated NSCs (aNSCs) and, subsequently, early intermediate progenitor cells (eIPCs). Using the ‘pseudotime’ concept to analyze the single-cell transcriptome data, Shin et al. [39] and Llorens-Bobadilla et al. [63] charted a continual developing trajectory for this early neurogenesis process. Like the work of Treutlein et al. [49], these two studies again demonstrate that the single-cell RNA-seq approach can provide a snapshot of the transcriptome dynamics of a developmental process if reasonable numbers of individual cells of the population are sequenced at a given time point.

The continual self-renewal of the intestinal epithelium is another well-established model for studying adult stem cells. Lgr5-positive cells positioned at crypt bottoms serve as the stem cells that fuel the self-renewal process. Grun et al. [37] sequenced nearly 200 green fluorescent protein (GFP)-marked Lgr5-positive cells, and found that these cells formed a single large homogenous population with a few outliers, which indicates a distinct population structure different from that of HSCs and NSCs.

Perturbation of the stem cell populations under non-physiological conditions has also been studied. Llorens-Bobadilla et al. [63] analyzed NSCs in ischemic brain injury. In NSCs under physiological conditions, these authors identified a transition from dormant NSCs to primed-quiescent NSCs and then activated NSCs. In injured NSCs, the authors found that the proportion of dormant NSCs prominently decreases, whereas the primed-quiescent and activated NSCs greatly increase. In another study, Kowalczyk et al. [60] compared young and old mice and found that ageing is associated with a decrease in the length of the G_1_ phase of the LT-HSCs, which should be linked to LT-HSC accumulation in older mice. In addition, they found that the transcriptome states of the ageing HSCs are inversely correlated with their differentiation states, such that the old ST-HSCs are similar to the young LT-HSCs [60]. Tsang et al. [61] investigated the knockout phenotype of the transcription factor Bcl11a and found abnormal proliferation and selective elimination of lymphoid-competent HSCs in Bcl11a-knockout HSCs [61]. Together, these studies demonstrate that single-cell RNA-seq can provide rich information on the structure of a stem cell population and its behavior under different conditions, and offer great insight into the function of tissue-specific stem cells.

### Cancer stem cells

Cancer tissue usually contains sub-populations of cells that have strong phenotypic and functional heterogeneity. The cancer stem cell (CSCs) concept holds that there is a sub-population of highly malignant stem cells at the top of the tumor cell hierarchy. The existence of these CSCs, however, is still controversial in many cancer types. Single-cell RNA-seq has the potential to help identify these cells and, more generally, to provide new insight into complex intra-tumoral heterogeneity. Patel et al. [57] sequenced 672 single cells from five glioblastoma samples. Each tumor showed high intra-tumoral cell heterogeneity in many aspects, including copy number variations as well as cell cycle, hypoxia, and immune response. By examining a set of ‘stemness’ genes, Patel et al. identified continuous, rather than discrete, stemness-related expression states among the individual cells of all five tumors, reflecting the complex stem cell states within a primary tumor. Even though there have been only a few studies addressing the question of tumor transcriptome heterogeneity down to the single-cell resolution [57, 72, 73], a more complete and accurate view of heterogeneity in various cancer types, including the characteristics of the CSC, is expected to be obtained in the near future.

## Single-cell epigenome sequencing technologies

The development, maintenance and differentiation of a stem cell are orchestrated by epigenetic modifications of its genome, including covalent modifications of genomic DNA and histones. Cell-to-cell epigenetic variation is an important layer of cell heterogeneity necessary for the transcriptional regulation of gene expression. Of particular interest will be the epigenome heterogeneity that underlies the transcriptome heterogeneity of cell populations such as the pluripotent and adult stem cells described above. In addition, how these heterogeneities are associated with changes in chromosome conformation in individual cells is not yet known. Conventional genome-wide epigenetic methods require millions of cells and cannot identify epigenetic heterogeneity among different individual cells, but recent studies have made great efforts in developing technologies to perform single-cell epigenome analysis (Table 2).

**Table 2:** Summary of single-cell epigenome sequencing technologies

Epigenetic marks	Assays	Strategies	Coverage	Reference
5mC	scRRBS	RRBS	0.5–2 M CpG sites	[74]
5mC	scBS	PBAT	0.5–10 M CpG sites	[75]
5mC	scWGBS	PBAT-like	0.5–10 M CpG sites	[76]
Chromatin accessibility	scATAC-seq	ATAC-seq	Average 73,000 unique fragments mapping to genome	[84]
Chromatin accessibility	scATAC-seq	ATAC-seq	50–6000 DHS sites	[85]
Chromatin accessibility	scDNase-seq	DNase-seq	Average 317,000 unique fragments and 38,000 DHS	[86]
Chromatin structure	Single-cell Hi-C	Hi-C	Not available	[87]
Chromatin structure	Single-cell DamID	DamID	Not available	[88]
Histone modification	Drop-ChIP	Droplet-based ChIP-seq	1000 H3K4me2 peaks	[89]

*5mC* 5-methylcytosine, *ATAC* assay for transposase-accessible chromatin, *BS* bisulfite sequencing, *ChIP* chromatin immunoprecipitation, *DamlD* Dam identification, *DHS* DNase I hypersensitive sites, *MALBAC* multiple annealing and looping-based amplification cycles, *PBAT* post-bisulfite adaptor tagging, *RRBS* reduced representation bisulfite sequencing, *sc* single-cell, *WGBS* whole-genome bisulfite sequencing

### DNA modifications

DNA methylation is the major DNA modification in the mammalian genome and plays important roles in many developmental processes. Recently, single-cell DNA methylome sequencing methods have been reported by our group and others [74–76]. Our method (scRRBS) is based on the reduced representation bisulfite sequencing (RRBS) strategy [77], whereas the methods of Smallwood et al. (scBS-seq) [75] and Farlik et al. (scWGBS) [76] are based on a post-bisulfite adaptor tagging (PBAT) approach [78]. Using these methods, we have charted the DNA methylation landscapes of human and mouse pre-implantation development, as well as human PGC development [62, 74, 79]. These and other studies have comprehensively characterized the two global DNA demethylation waves that occur during mammalian pre-implantation and PGC development at the genome-scale and the single-base resolution [80–82]. These studies have shown that human PGCs at about 10 to 11 weeks after gestation have lower methylation levels (6–8 %) than other types of cells including blastocysts (~40 %). This serial hypomethylated DNA methylome dataset of human PGCs in vivo can be used as a standard reference for assessing the quality of PGC-like cells differentiated from hESCs or human induced pluripotent stem cells (hiPSCs) in vitro. Smallwood et al. [75] demonstrated that integration of just 12 single oocyte scBS-seq datasets can largely recover the major pattern of their entire DNA methylome. Although successful, the present single-cell DNA methylome sequencing methods have much sparser coverage than bulk methods, and thus have much room for improvement. In addition to DNA methylation, recent studies have uncovered hydroxymethylation (5hmC) as well as 5-formylcytosine (5fC) and 5-carboxylcytosine (5caC) modifications on genomic DNAs [83]. Although whole genome scale methods for detecting these DNA modifications on bulk cells have been established, methods at the single-cell level still await development in the near future.

### Chromatin accessibility and structure

Genomic methods for assessing the chromatin accessibility of bulk cell populations have been effective for identifying active regulatory elements. Several recent studies have adapted these methods to single-cell resolution. The methods of Buenrostro et al. [84] and Cusanovich et al. [85] (scATAC-seq) are based on ATAC-seq (assay for transposase-accessible chromatin) and rely on the ability of the prokaryotic Tn5-transposase to insert preferentially into accessible chromatin regions in the genome. The method used by Jin et al. [86] is based on the more conventional DNase sequencing approach (scDNase-seq). scDNase-seq appears to detect more open chromatin regions per individual cell than scATAC-seq. In addition, chromosome structure capture technologies have recently been adapted to single cell analysis [87, 88]. These methods, which have been shown to distinguish correctly between ESCs and other cell types at different chromatin state layers [86], should be applied to dissect the heterogeneity of chromatin states of stem cell populations in the near future.

### Histone modifications

Histone modifications play essential roles in the regulation of gene expression in stem cells. Chromatin immunoprecipitation followed by sequencing (ChIP-seq) is a widely used method for mapping histone modifications at the whole-genome scale. Rotem et al. [89] recently adapted ChIP-seq to a single-cell analysis by combining droplet and barcoding strategies (Drop-ChIP). A stringent negative control using a non-specific IgG antibody was not performed side-by-side for murine embryonic fibroblasts (MEFs) or ESCs, however, leaving the potential non-specific noise in their single-cell ChIP-seq dataset unresolved. Drop-ChIP is able to detect only approximately 1000 H3K4me3 peaks per cell, corresponding to a peak detection sensitivity of approximately 5 %. Nevertheless, the method is capable of separating mouse ESCs into three sub-populations that have distinct H3K4me2 signals over loci bound by pluripotency-associated transcription factors such as Oct4, Sox2, and Nanog, and differentiation-associated transcription factors such as FoxA2, as well as epigenetic repressors including Polycomb and CoREST. The first group of cells has the highest signal for these pluripotency signature genes, the second group has intermediate signals, and the third group has the lowest signals, while H3K4me2 signals for differentiation and epigenetic repressor signature genes are reversed. Thus, these sub-populations may have distinct chromatin states that are related to pluripotency and differentiation priming. This finding implicates a new layer of cell heterogeneity in the epigenome of ESCs. Further improvement of single-cell epigenome sequencing technologies will provide a deeper understanding of the cell heterogeneity of chromatin states in ESCs and other types of stem cells.

## Single-cell genome sequencing technologies

The genomes of individual cells carry another layer of information that is useful in revealing the development and heterogeneity of a stem cell population: the cell lineage. During development, one stem cell gives rise to many specialized cells through continuous cell division and differentiation. During each cell division, replication errors may occur. Although such errors (replication mutations) occur at an extremely low frequency in normal mammalian cells (0–1 mutations per cell division), any replication mutations that are detected in individual progeny cells can be used to trace the developmental lineage of those cells. A cell lineage tree, such as the detailed lineage tree that has been illustrated for *Caenorhabditis elegans*, can greatly help to illustrate a developmental process.

To detect replication mutations in individual cells, a single-cell whole-genome amplification is necessary in order to get enough material for sequencing analysis. This can be accomplished using methods that include degenerate oligonucleotide-primed polymerase chain reaction (DOP-PCR) [90], multiple displacement amplification (MDA) [91], MALBAC [92], microfluidics-based MDA [93–95] and MDA for G_2_/M nuclei (Nuc-seq) [96, 97] (Table 3). Detailed and elegant reviews of these methods can also be seen elsewhere [98, 99].

**Table 3:** Summary of single-cell genome sequencing technologies

Assays	Strategies	Principles	Reference(s)
DOP-PCR	Degenerate oligonucleotide-primed PCR	Exponential	[90]
MDA	Phi 29 DNA polymerase-based MDA	Exponential	[91]
MALBAC	Multiple annealing and looping-based amplification cycles	Quasi-linear	[92]
Microfluidic MDA	MDA in a microfluidic chamber	Exponential	[93]
MIDAS	MDA in hundreds to thousands of nanoliter wells	Exponential	[94]
eWGA	MDA in millions of picoliter droplets	Exponential	[95]
Nuc-seq	MDA for single cells in S phase	Exponential	[96, 97]

*DOP* degenerate oligonucleotide-primed, *eWGA* emulsion whole-genome amplification, *MALBAC* multiple annealing and looping-based amplification cycles, *MDA* multiple displacement amplification, *PCR* polymerase chain reaction

Single-cell genome sequencing has been applied to human germ cells for sperm and oocytes to study meiotic recombination, aneuploidy, and the mutation rate of these cells [93, 100, 101]. These studies have generated the first personal recombination maps of individual men and women and have detected aneuploidy during human gametogenesis [93, 100, 101]. Behjati et al. [102] have also applied genome sequencing for lineage tracing of the development of normal cells. In this study, the early cell lineage and the contribution of these early cells to adult tissues were elucidated by whole-genome sequencing of 25 single-cell-derived organoid lines from the mouse gut and prostate. Single-cell whole-genome sequencing has also been used to study tumor cells. Clonal evolution of a tumor can be elucidated on the basis of the copy number variation (CNV) and single-nucleotide variation (SNV) of single tumor cells [96]. How to authenticate a SNV accurately within a single cell with essentially no false-positive calls remains a challenge. Future improvement of single-cell whole-genome-sequencing technologies will help resolve this issue and will promote the application of this technology for the lineage tracing of stem cells by comprehensively identifying genomic variations within each single stem or differentiated cell in normal or cancerous tissue.

## Conclusions

Despite the fact that single-cell sequencing methods have been widely applied to dissecting the heterogeneity of stem cells, all of the currently available single-cell omic sequencing technologies are clearly not ideal. There exist significant technical noise and amplification errors, and they provide relatively low coverage when compared to bulk sequencing methods. This is expected, as the whole field is still in its infancy beginning only 7 years ago. From this perspective, it is amazing that the single-cell omic sequencing field has already had such great influence and has contributed so tremendously to numerous biological fields. There is huge room for additional development and improvement of the technologies.

Amplification error is a crucial parameter and an issue that limits the accuracy of current single-cell omic sequencing technologies, all of which are based on the pre-amplification of the nucleic acids in individual cells before deep sequencing. After amplification, the single cell being analyzed is already ‘destroyed’; thus, the results cannot be verified in the same individual cell. Some reports use Sanger sequencing to re-sequence the amplified product from the same individual cell for selected loci at which point mutations have been called. Nevertheless, this strategy can detect only the next generation sequencing errors, leaving the single-cell amplification errors concealed and untestable. The other strategy is to use several cells to verify each other and to count only the SNVs that are called in three or more individual cells [92, 96]. Unfortunately, this approach is possible only for cells that can be cultured and amplified at a clonal level in vitro, which is very difficult, if not impossible, for the majority of types of primary cells. If such cell preparation is not possible, this approach will remove the real SNVs that are unique to an individual cell and will severely limit the applications of single-cell omic sequencing technologies. An ideal single-cell genome-sequencing technology would accurately identify both common and ‘private’ SNVs within an individual cell without any false positives resulting from amplification errors. We propose that, in the near future, better single-cell omic sequencing technologies should permit several repeated measurements of the original copy of the nucleic acids within an individual cell. In this way, the amplification errors of sequencing a single cell could be accurately and directly evaluated and determined within the same cell. This would also permit the authentic mutation in an individual cell to be firmly called and verified with essentially no false positives.

It is also important to develop a full set of new bioinformatics tools that are specifically designed for analyses of single-cell omic datasets. These bioinformatics tools should carefully consider both the cons of the single-cell omic datasets, such as high technical noise and high false-negative rates, and the pros of these datasets, such as high sampling numbers and UMIs or spike-in based absolute counting. Despite being valuable, current ‘pseudotime’ analyses have problems in resolving some of the intermediate states during differentiation, especially when these states are dramatically different from both the earlier stem cell state and the later committed state. This is due to the fact that single-cell transcriptome analysis by its nature provides only a snapshot of the gene expression profile for each individual cell, which is an intrinsic disadvantage of this technique when compared with time-lapse imaging methods. As the technique can offer a whole-genome-scale gene expression profile, and because the gene-expression changes in an individual cell at the whole-transcriptome scale can usually be assumed to be ‘continual’ and traceable within a short time interval, one possible resolution for the ‘snapshot’ problem is to sample the cell population much more intensely, ideally every hour or so. Including the following assumption into the pseudotime algorithms is also likely to be helpful: the later differentiation time point will very probably contain differentiation-delayed residual stem cells of the earlier time point. By contrast, the earlier time point is very unlikely to contain fully differentiated cells. For example, during ESC differentiation into liver cells, functional liver cells are very unlikely to be found in the population after just 1 or 2 days; but in the several-week differentiated cell population that contains functional liver cells, it will still be possible to find some residual stem-like cells. Adding this constraint will probably help to resolve the true differentiation pathway of stem cells.

Single-cell multiple omics sequencing technologies have also been developed recently. These methods are capable of simultaneously obtaining information from a single cell on the transcriptome and genome (G&T-seq) [103], or on the transcriptome and DNA methylome (scM&T-seq) [104], or even on all three of these omics (genome, DNA methylome, and transcriptome; scTrio-seq) [73]. New methods covering more layers of different omics are expected to emerge in the near future. These methods are invaluable for elucidating the relationship between different layers of omics in an individual cell. When they become routinely available, permitting the precise recovery of genome, epigenome and transcriptome information from the same individual cell, an ideal approach would be to use single-cell genome sequencing data to perform lineage tracing to reconstruct the pedigree of the cells during stem cell differentiation in vivo. Then, transcriptome data from these cells could be analyzed and used to identify different cell types or sub-populations in the complex tissue. The epigenome information from the same set of single cells could be used subsequently to investigate how different epigenetic layers regulate transcription. Finally, to build a causal relationship between genotype and phenotype, it will be ideal to knockout key component genes for stem cells in vivo using gene-editing technologies. Single-cell multiple omics sequencing at serial time points during the development and differentiation process of stem cells could then be used to reconstruct the core gene regulation network within each individual cell during the differentiation process. The phenotype–genotype relationship for each gene within each individual cell, or between different individual cells, will finally permit us to understand thoroughly the complexity and beauty of the gene regulation network under both physiological and pathological conditions, and will provide us with new insights into the biological basis of human development and diseases.

## Abbreviations

AT: alveolar type
ATAC: assay for transposase-accessible chromatin
BS: bisulfite sequencing
ChIP: chromatin immunoprecipitation
CSC: cancer stem cell
EPI: pluripotent epiblast
ESC: embryonic stem cell
hESC: human embryonic stem cell
HSC: hematopoietic stem cell
ICM: inner cell mass
LT-HSC: long-term reconstituting hematopoietic stem cell
MALBAC: multiple annealing and looping-based amplification cycles
MDA: multiple displacement amplification
mESC: mouse embryonic stem cell
NSC: neural stem cell
PCA: principal component analysis
PCR: polymerase chain reaction
PE: primitive endoderm
PGC: primordial germ cell
RRBS: reduced representation bisulfite sequencing
sc: single-cell
seq: sequencing
SNV: single-nucleotide variation
ST-HSC: short-term reconstituting hematopoietic stem cell
TE: trophectoderm
UMI: unique molecular identifier
WGBS: whole-genome bisulfite sequencing
